# Supplementary material for: Benchmarking of methods for DNA methylome deconvolution
Source: Nat Commun. 2024 May 16;15:4134. doi: 10.1038/s41467-024-48466-z (PMC11099101; doi:10.1038/s41467-024-48466-z)
Supplement: Supplementary file 1 — Supplementary Figs. [file 41467_2024_48466_MOESM1_ESM.pdf]

# Benchmarking of Methods for DNA Methylome Deconvolution

## Supplementary Figures

### Supplementary Figure 1

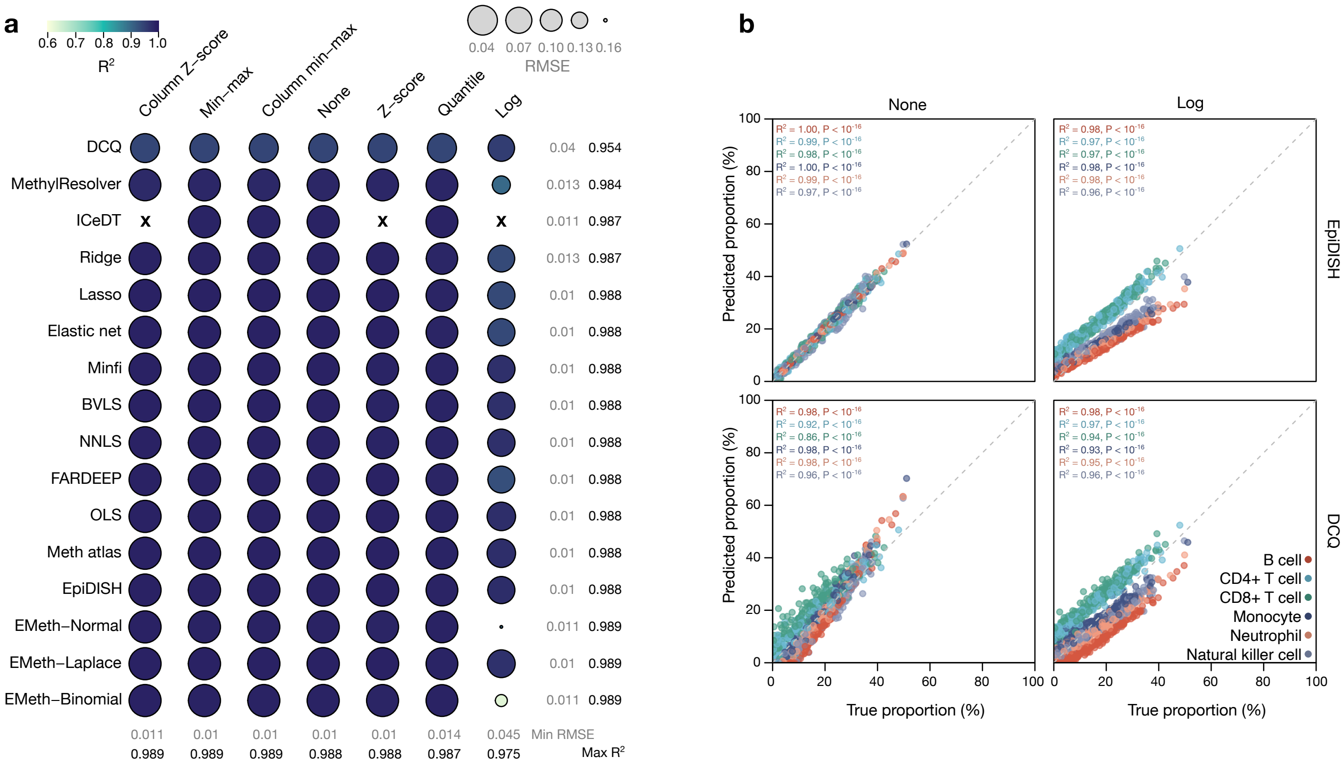


**Supplementary Figure 1 | Deconvolution on reference-based in silico mixtures. a.** Performance of deconvolution on 200 in silico mixtures. Algorithm-normalization combinations are visualized as circles. Spearman's $R^2$ represented by color, root mean squared error represented by size. Rows show deconvolution algorithms, columns show normalization methods. 'X' symbol represents missing values. **b.** Scatter plots showing true proportions (x-axis) and predicted proportions (y-axis) in percentages for the best performing (left-upper) and worst performing (right-lower) algorithm-normalization combinations on reference-based 200 in silico mixtures. $R^2$ and p-values were calculated using Spearman's rank correlation test. Source data are provided as a Source Data file.

## Supplementary Figure 2

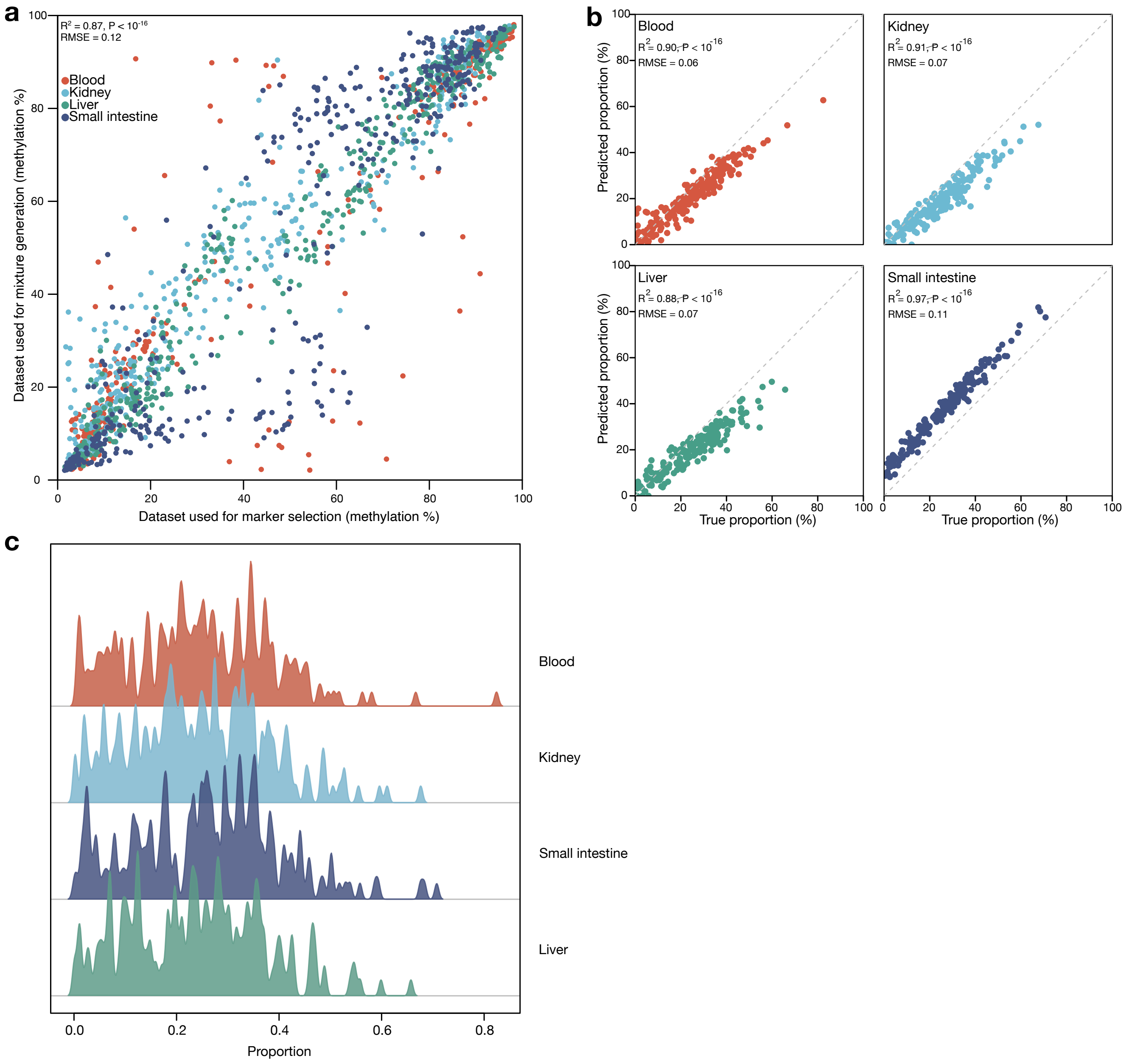


**Supplementary Figure 2 | Correlation of methylation rates between reference and validation 450K tissue datasets. a.** Scatter plot showing mean methylation ratios for reference (x-axis) and validation (y-axis) datasets in percentages at marker CpGs (n = 400) on in silico 450K tissue data. **b.** Scatter plots showing true proportions (x-axis) and predicted proportions (y-axis) in percentages on 200 in silico mixtures for all tissues using quantile normalization combined with ridge deconvolution. **c.** Ridgeline plot showing distributions of cell proportions per tissue. $R^2$ and p-values were calculated using Spearman's rank correlation test. Source data are provided as a Source Data file.

## Supplementary Figure 3

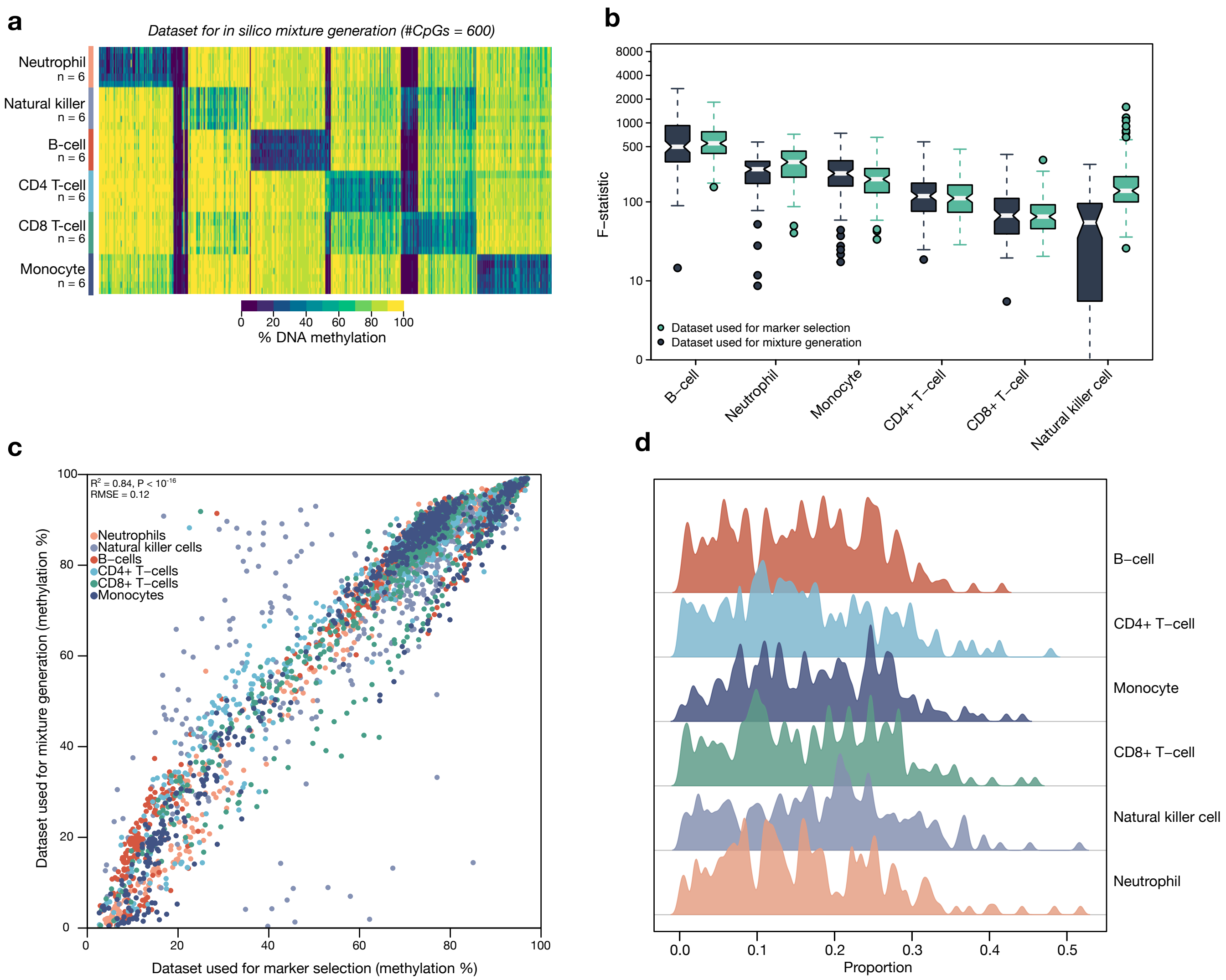


**Supplementary Figure 3 | Comparison of marker CpGs between reference and validation 450K immune cell datasets. a.** Matrix of marker CpGs (n = 600) used for building immune cell methylation in silico mixtures of 450K immune cell data. Samples for six cell types were included: neutrophil (n = 6), natural killer cell (n = 6), B-cell (n = 6), CD4+ T-cell (n = 6), CD8+ T-cell (n = 6) and monocyte (n = 6). **b.** Boxplots showing F-statistics for all cell types, at their respective marker CpGs, between reference and validation datasets. The boxplots present median values and quartiles, whiskers the minimum and maximum values, and dots the individual data points. **c.** Scatter plot showing mean methylation ratios for reference (x-axis) and validation (y-axis) datasets in percentages at marker CpGs on in silico 450K immune cell data. $R^2$ and p-values were calculated using Spearman's rank correlation test. **d.** Ridgeline plot showing distributions of cell proportions per cell type. Source data are provided as a Source Data file.

# Supplementary Figure 4

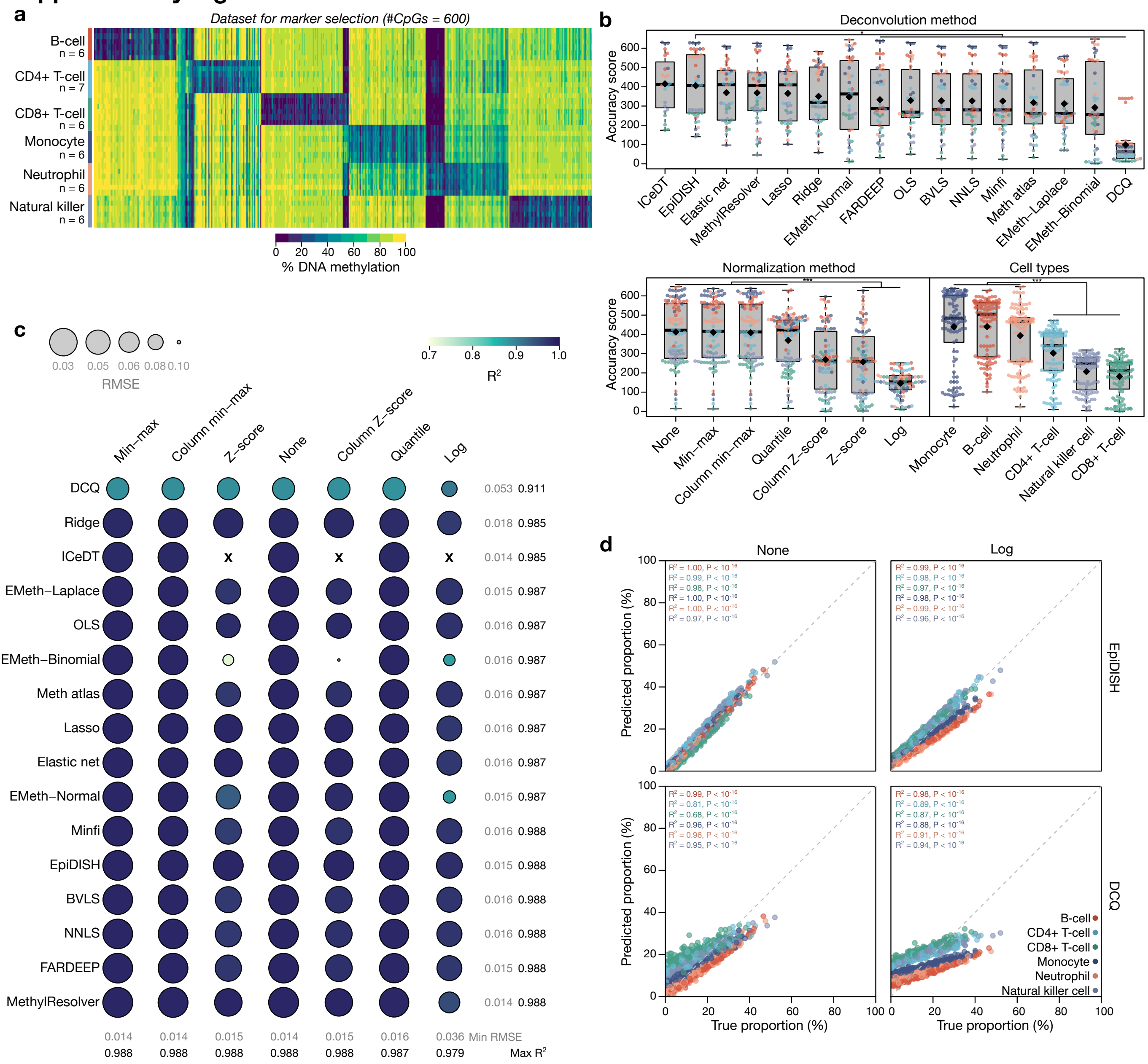

**Supplementary Figure 4 | Deconvolution of immune cell types on EPIC data using CpGs selected on 450K data. a.** Matrix of 450K marker CpGs (n = 600) used for building immune cell methylation reference of EPIC data. Samples for six cell types were included: neutrophil (n = 6), natural killer cell (n = 6), B-cell (n = 6), CD4+ T-cell (n = 7), CD8+ T-cell (n = 6) and monocyte (n = 6). **b.** Deconvolution accuracy represented as boxplots showing accuracy scores for the different deconvolution methods, normalization methods and cell types on 200 in silico mixtures. Black diamond shapes represent median values, colors represent cell types. The boxplots present median values and quartiles, whiskers the minimum and maximum values, and dots the individual data points. P-values were determined using two-sided FDR-adjusted Dunn's tests. * = $P < 0.05$, ** = $P < 0.01$, *** = $P < 0.001$. **c.** Performance of deconvolution on 200 in silico mixtures for all algorithm-normalization combinations represented as circles. Algorithm-normalization combinations are visualized as circles. Spearman's $R^2$ represented by color, root mean squared error represented by size. Rows show deconvolution algorithms, columns show normalization methods. **d.** Scatter plots showing true proportions (x-axis) and predicted proportions (y-axis) in percentages for the best performing (left-upper) and worst performing (right-lower) deconvolution and normalization algorithms on 200 in silico mixtures. $R^2$ and p-values were calculated using Spearman's rank correlation test. 'X' symbol represents missing values. Source data are provided as a Source Data file.
Exact p-values are added in the Source Data file.

# Supplementary Figure 5

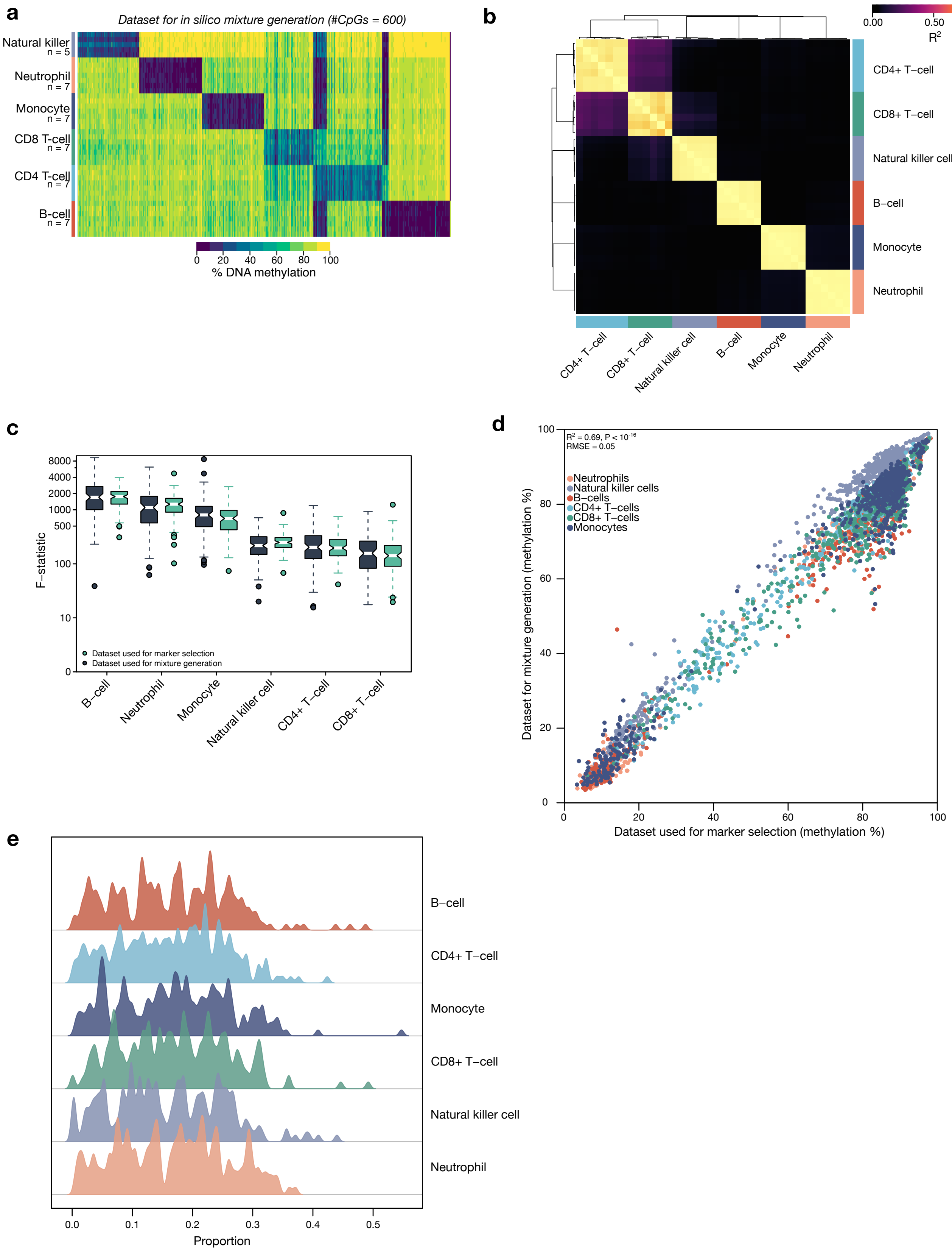

**Supplementary Figure 5 | Comparison of marker CpGs between reference and validation EPIC immune cell datasets. a.** Matrix of marker CpGs (n = 600) used for building immune cell methylation in silico mixtures of EPIC immune cell data. Samples for six cell types were included: neutrophil (n = 7), natural killer cell (n = 5), B-cell (n = 7), CD4+ T-cell (n = 7), CD8+ T-cell (n = 7) and monocyte (n = 7). **b.** Correlation matrix showing $R^2$ values of methylation values at marker loci between samples of the dataset used for marker selection. $R^2$ values were calculated using Spearman's rank correlation test. **c.** Boxplots showing F-statistics for all cell types, at their respective marker CpGs, between reference and validation datasets. The boxplots present median values and quartiles, whiskers the minimum and maximum values, and dots the individual data points. **d.** Scatter plot showing mean methylation ratios for reference (x-axis) and validation (y-axis) datasets in percentages at marker CpGs on in silico EPIC immune cell data. $R^2$ and p-values were calculated using Spearman's rank correlation test. **e.** Ridgeline plot showing distributions of cell proportions per cell type. Source data are provided as a Source Data file.

# Supplementary Figure 6

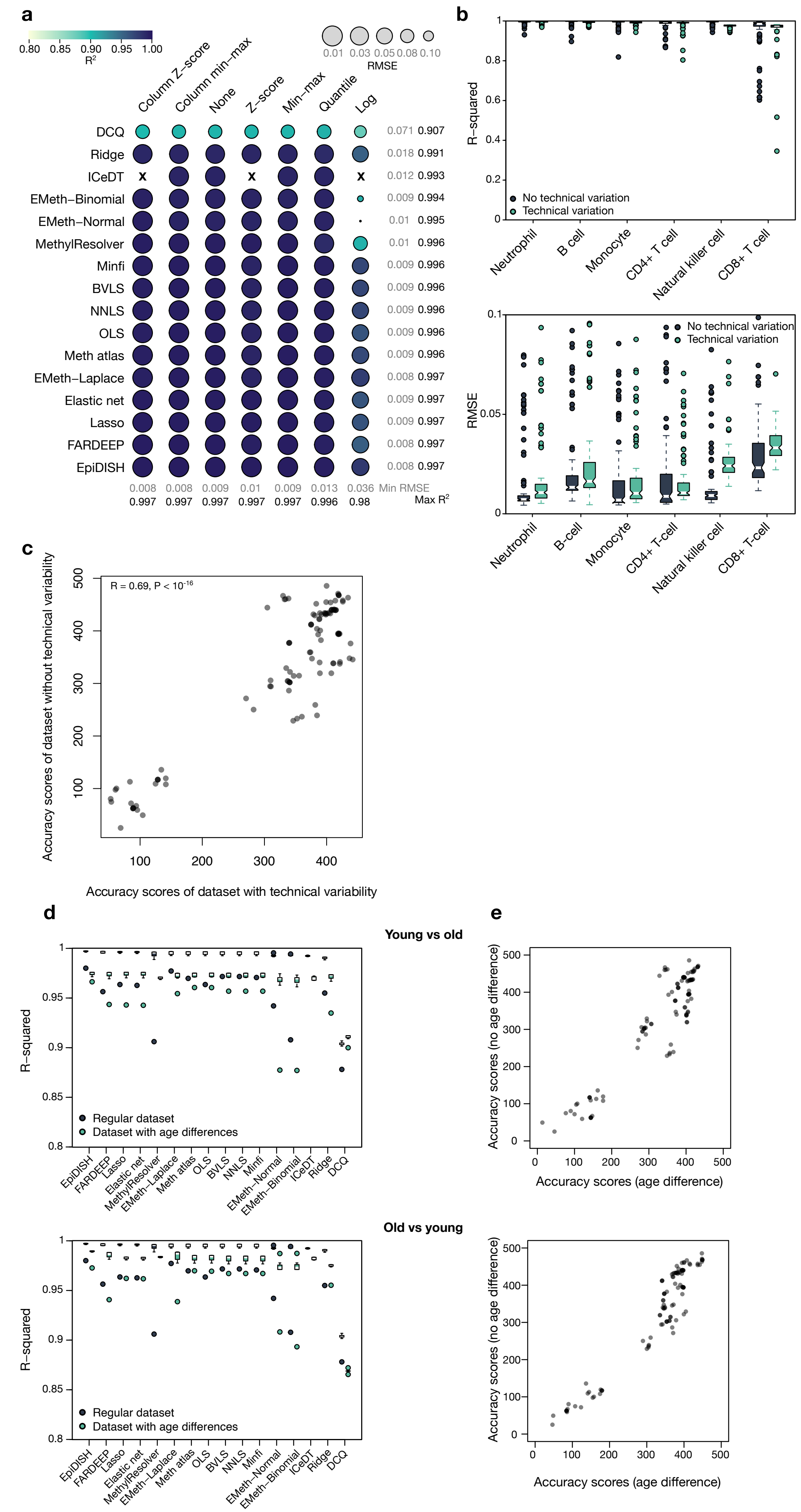

**Supplementary Figure 6 | Impact of inter-dataset variation on deconvolution performance. a.** Performance of deconvolution on 200 in silico mixtures for all algorithm-normalization combinations represented as circles. Algorithm-normalization combinations are visualized as circles. Spearman's $R^2$ represented by color, root mean squared error represented by size. Rows show deconvolution algorithms, columns show normalization methods. **b.** Boxplots showing Spearman's $R^2$ and RMSE for 200 in silico mixtures comparing datasets with and without inter-dataset variation. The boxplots present median values and quartiles, whiskers the minimum and maximum values, and dots the individual data points. **c.** Scatter plot showing accuracy scores for all algorithm-normalization combinations from datasets with (x-axis) and without (y-axis) technical variability. $R^2$ and p-values were calculated using Spearman's rank correlation test. **d.** Scatterplot showing correlation between accuracy scores for algorithm-normalization combinations on in silico mixtures generated from a dataset with minimal technical variation compared to the reference (y-axis) and datasets with significant age difference compared to the reference (x-axis). The boxplots present median values and quartiles, whiskers the minimum and maximum values, and dots the individual data points. **e.** Boxplots showing $R^2$ values for each deconvolution algorithm on in silico mixtures generated from a dataset with minimal technical variation compared to the reference and from a dataset with significant age difference compared to the reference. Source data are provided as a Source Data file.

## Supplementary Figure 7

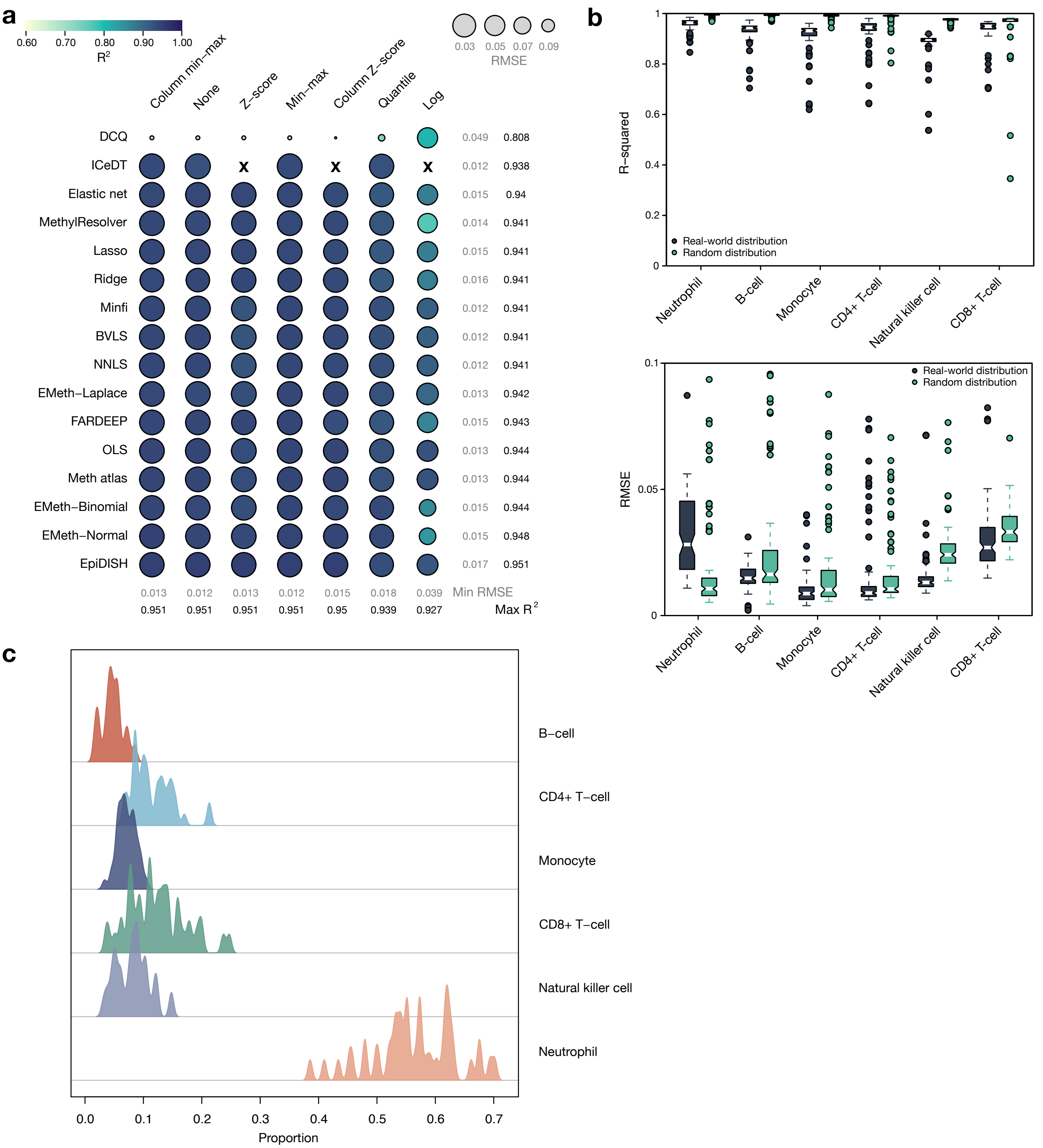


**Supplementary Figure 7 | Impact of proportion distribution on deconvolution performance. a.** Performance of deconvolution on 200 in silico mixtures for all algorithm-normalization combinations represented as circles. Algorithm-normalization combinations are visualized as circles. Spearman's $R^2$ represented by color, root mean squared error represented by size. Rows show deconvolution algorithms, columns show normalization methods. 'X' symbol represents missing values. **b.** Boxplots showing Spearman's $R^2$ and RMSE for 200 in silico mixtures comparing datasets with proportion distributions generated from real-life datasets or uniform distributions. The boxplots present median values and quartiles, whiskers the minimum and maximum values, and dots the individual data points. **c.** Ridgeline plot showing distributions of cell proportions per cell type in in silico mixtures generated with real-life proportions. Source data are provided as a Source Data file.

# Supplementary Figure 8

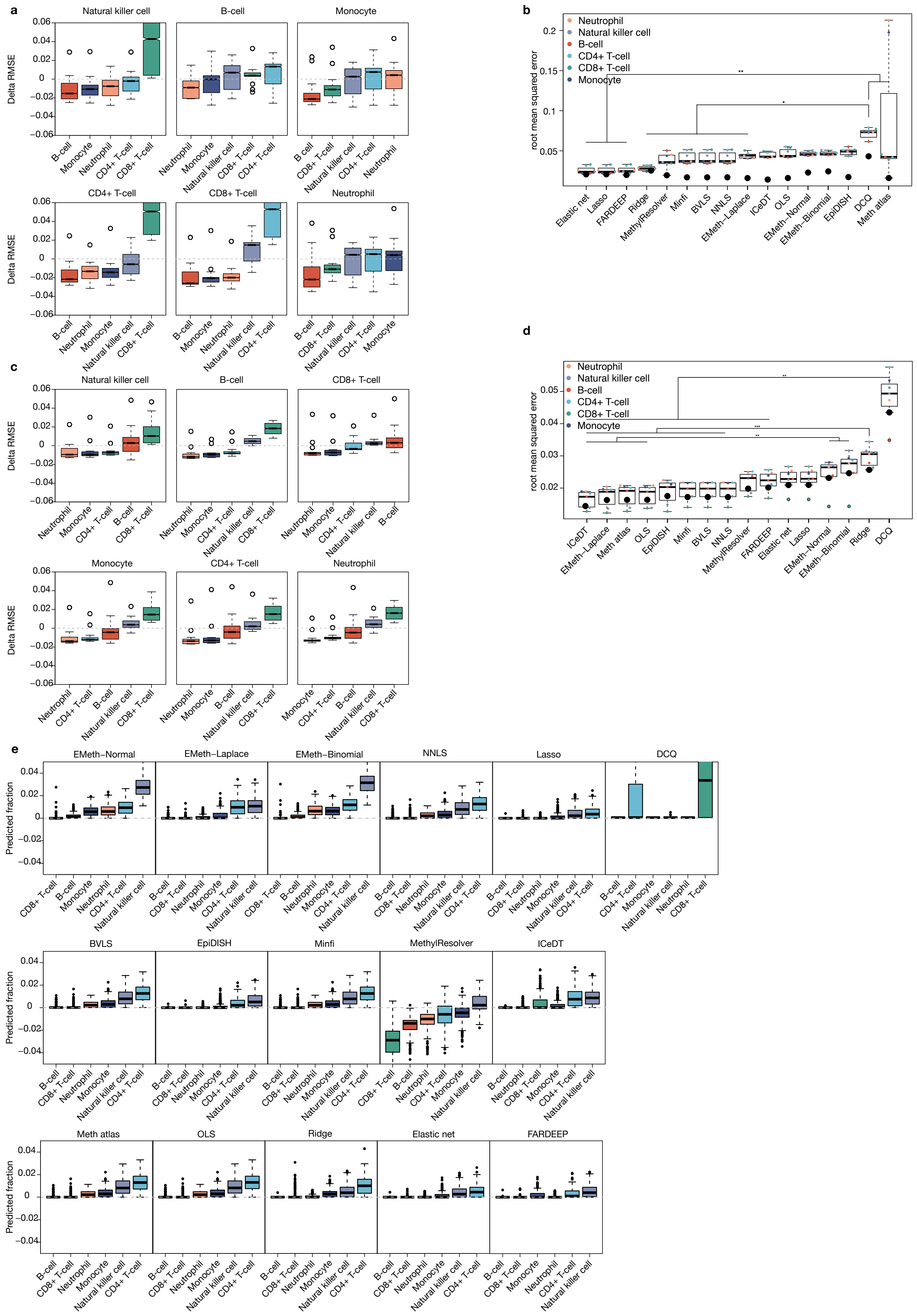

**Supplementary Figure 8 | Impact of incomplete or over-extensive reference on deconvolution. a.** Boxplots showing root mean squared error (RMSE) values while leaving one cell type out of the reference, subtracted from root mean squared error values in the normal setting (i.e., equal number of cell types in reference and mixture). Titles represent removed cell types. X-axis represents the deconvolved cell types. **b.** Boxplot showing RMSE values for each deconvolution method while leaving one cell type out of the reference. Black dots represent RMSE in the normal setting, colors represent the cell type that was left out. **c.** Boxplots showing RMSE values while leaving one cell type out of the mixture, subtracted from root mean squared error values in the normal setting. Titles represent removed cell types. X-axis represents the deconvolved cell types. **d.** Boxplot showing RMSE values for each deconvolution method while leaving one cell type out of the mixture. Black dots represent RMSE in the normal setting, colors represent the cell type that was left out. **e.** Boxplots showing predicted fractions for cell types that are not present in the mixture for each deconvolution method separately. P-values were determined using two-sided FDR-adjusted Dunn's tests. * = $P < 0.05$, ** = $P < 0.01$, *** = $P < 0.001$. The boxplots present median values and quartiles, whiskers the minimum and maximum values, and dots the individual data points. Source data are provided as a Source Data file. Exact p-values are added in the Source Data file.

# Supplementary Figure 9

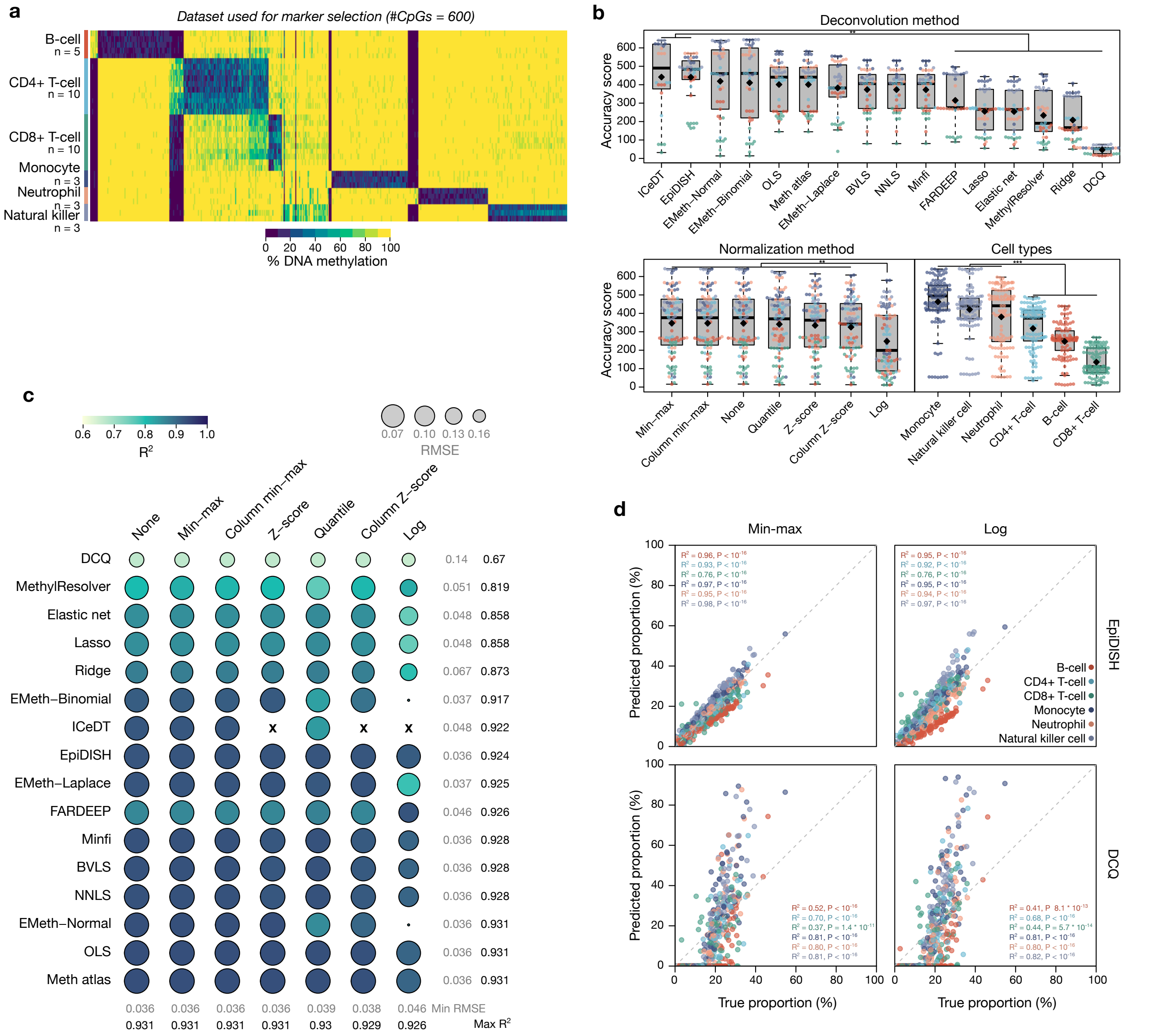


**Supplementary Figure 9 | Deconvolution of immune cell types on WGBS data using marker CpGs. a.** Matrix of marker CpGs (n = 600) used for building immune cell methylation reference of WGBS data. Samples for six cell types were included: neutrophil (n = 3), natural killer cell (n = 3), B-cell (n = 5), CD4+ T-cell (n = 10), CD8+ T-cell (n = 10) and monocyte (n = 3). **b.** Deconvolution accuracy showing RMSE values for the different deconvolution methods, normalization methods and cell types on 200 in silico mixtures. Black diamond shows median, colors represent cell types. The boxplots present median values and quartiles, whiskers the minimum and maximum values, and dots the individual data points. P-values were determined using two-sided FDR-adjusted Dunn's tests. * = P < 0.05, ** = P < 0.01, *** = P < 0.001. **c.** Performance of deconvolution on 200 in silico mixtures. Algorithm-normalization combinations are visualized as circles. Spearman's $R^2$ represented by color, root mean squared error represented by size. Rows show deconvolution algorithms, columns show normalization methods. **d.** Scatter plots showing true proportions (x-axis) and predicted proportions (y-axis) in percentages for the best performing (left-upper) and worst performing (right-lower) deconvolution and normalization algorithms on 200 in silico mixtures. $R^2$ and p-values were calculated using Spearman's rank correlation test. 'X' symbol represents missing values. Source data are provided as a Source Data file. Exact p-values are added in the Source Data file.

## Supplementary Figure 10

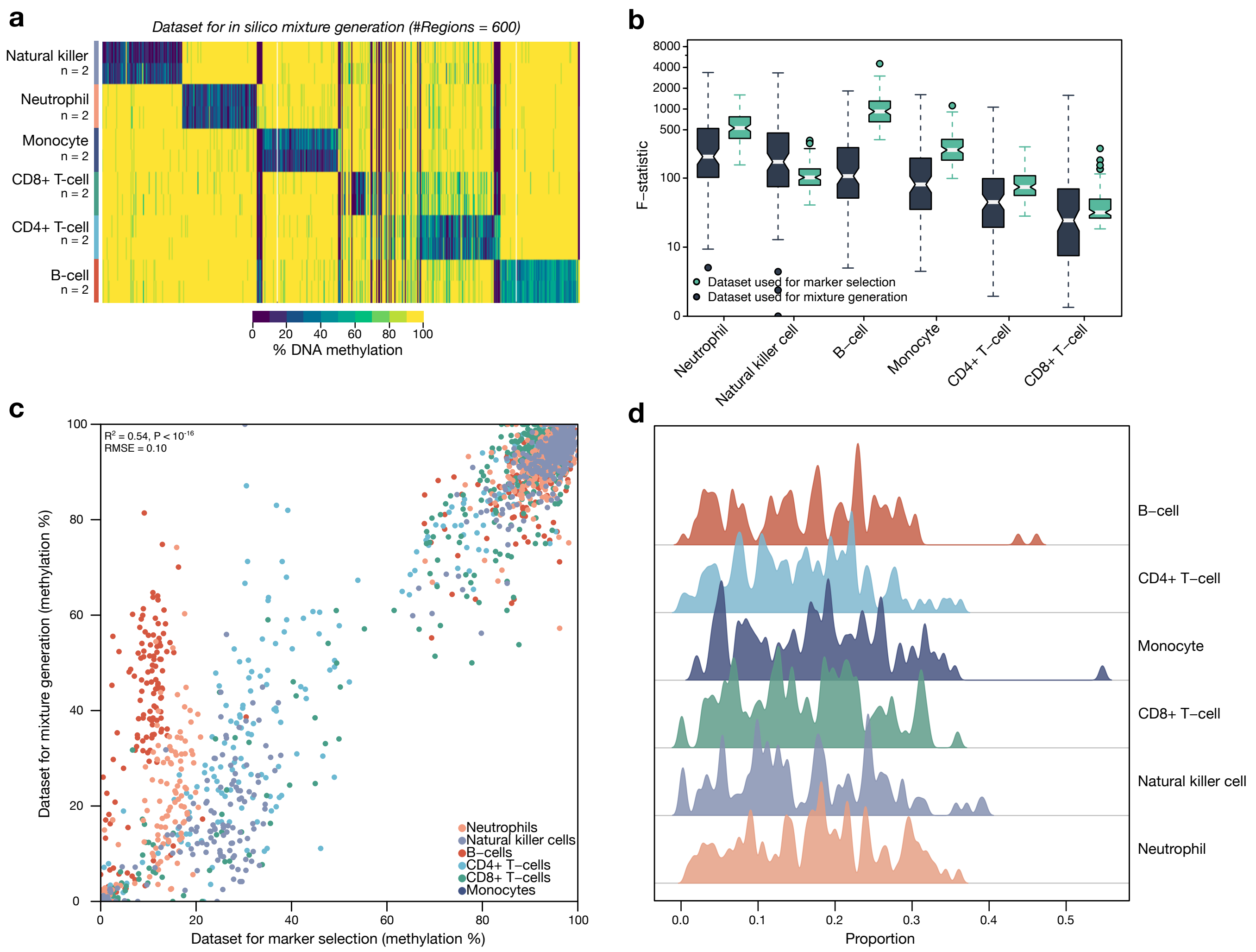


**Supplementary Figure 10 | Comparison of marker regions between reference and validation WGBS immune cell datasets. a.** Matrix of marker regions (n = 600) used for building immune cell methylation in silico mixtures of WGBS immune cell data. Samples for six cell types were included: neutrophil (n = 9), natural killer cell (n = 2), B-cell (n = 4), CD4+ T-cell (n = 8), CD8+ T-cell (n = 2) and monocyte (n = 2). Reads were compiled in 1 large pool per cell type, used during in silico mixture building. For visualization purposes, these were split into 2 parts to showcase methylation consistency. **b.** Boxplots showing F-statistics for all cell types, at their respective marker CpGs, between reference and validation datasets. The boxplots present median values and quartiles, whiskers the minimum and maximum values, and dots the individual data points. **c.** Scatter plot showing mean methylation ratios for reference (x-axis) and validation (y-axis) datasets in percentages at marker regions on in silico WGBS immune cell data. $R^2$ and p-values were calculated using Spearman's rank correlation test. **d.** Ridgeline plot showing distributions of cell proportions per cell type. Source data are provided as a Source Data file.
