# Supplementary material for: Benchmarking of methods for DNA methylome deconvolution
Source: Nat Commun. 2024 May 16;15:4134. doi: 10.1038/s41467-024-48466-z (PMC11099101; doi:10.1038/s41467-024-48466-z)
Supplement: Supplementary file 3 — Description of Additional Supplementary Files [file 41467_2024_48466_MOESM3_ESM.pdf]

Description of Additional Supplementary Files:

Supplementary Dataset 1: Normalization methods included for benchmarking.

Supplementary Dataset 2: Marker CpGs and regions included for building reference matrices.

Supplementary Dataset 3: Identifiers of datasets and corresponding proportions used for mixtures.

Supplementary Dataset 4: R2 values for algorithm-normalization combinations over all relevant experiments.

Supplementary Dataset 5: RMSE values for algorithm-normalization combinations over all relevant experiments.

Supplementary Dataset 6: JSD values for algorithm-normalization combinations over all relevant experiments.

Supplementary Dataset 7: Accuracy score values for algorithm-normalization combinations over all relevant experiments.
