# Supplementary material for: Benchmarking of methods for DNA methylome deconvolution
Source: Nat Commun. 2024 May 16;15:4134. doi: 10.1038/s41467-024-48466-z (PMC11099101; doi:10.1038/s41467-024-48466-z)
Supplement: Supplementary file 11 — Reporting Summary [file 41467_2024_48466_MOESM11_ESM.pdf]

nature portfolio

Corresponding author(s): Bernard Thienpont

Last updated by author(s): Apr 17, 2024

Reporting Summary

Nature Portfolio wishes to improve the reproducibility of the work that we publish. This form provides structure for consistency and transparency in reporting. For further information on Nature Portfolio policies, see our Editorial Policies and the Editorial Policy Checklist.

Statistics

For all statistical analyses, confirm that the following items are present in the figure legend, table legend, main text, or Methods section.

n/a	Confirmed	
☐	☒	The exact sample size (n) for each experimental group/condition, given as a discrete number and unit of measurement
☐	☒	A statement on whether measurements were taken from distinct samples or whether the same sample was measured repeatedly
☐	☒	The statistical test(s) used AND whether they are one- or two-sided<br>*Only common tests should be described solely by name; describe more complex techniques in the Methods section.*
☐	☒	A description of all covariates tested
☐	☒	A description of any assumptions or corrections, such as tests of normality and adjustment for multiple comparisons
☐	☒	A full description of the statistical parameters including central tendency (e.g. means) or other basic estimates (e.g. regression coefficient) AND variation (e.g. standard deviation) or associated estimates of uncertainty (e.g. confidence intervals)
☐	☒	For null hypothesis testing, the test statistic (e.g. F, t, r) with confidence intervals, effect sizes, degrees of freedom and P value noted<br>*Give P values as exact values whenever suitable.*
☒	☐	For Bayesian analysis, information on the choice of priors and Markov chain Monte Carlo settings
☒	☐	For hierarchical and complex designs, identification of the appropriate level for tests and full reporting of outcomes
☐	☒	Estimates of effect sizes (e.g. Cohen's d, Pearson's r), indicating how they were calculated

Our web collection on statistics for biologists contains articles on many of the points above.

Software and code

Policy information about availability of computer code

Data collection	For data collected from European Genome-Phenome Archive, pyEGA3 was used. No software was used for downloading Gene Expression Omnibus data.
Data analysis	Sequencing reads were trimmed with TrimGalore! (vü.6.6) to remove potential adapter contamination and to trim off the adaptase-induced addition of random nucleotides. Next, trimmed reads were aligned to the reference human genome GRCh37 using Bwameth (v0. 2 .5). Reads were deduplicated using Picard (v3.I.0) and methylation was called using MethylDackel (v0.6.0). Further analyses were performed using R (v 4.1.1) and Python (v3.8.II.).

For manuscripts utilizing custom algorithms or software that are central to the research but not yet described in published literature, software must be made available to editors and reviewers. We strongly encourage code deposition in a community repository (e.g. GitHub). See the Nature Portfolio guidelines for submitting code & software for further information.

Data

Policy information about availability of data

All manuscripts must include a data availability statement. This statement should provide the following information, where applicable:

- Accession codes, unique identifiers, or web links for publicly available datasets
- A description of any restrictions on data availability
- For clinical datasets or third party data, please ensure that the statement adheres to our policy

The data used in this study from the Gene Expression Omnibus (GEO) are available under the following accession codes: GSE59157, GSE50874, GSE61258, GSE61278, GSE73832, GSE50475, GSE48472, GSE84003, GSE71244, GSE65097, GSE35069, GSE77797, GSE110554, GSE103541, GSE129376 and GSE186458. The data used in this study from the European Genome-phenome Archive (EGA) are available under the following accession codes after data access authorization: EGAD00001000710, EGAD00001001189, EGAD00001001261, EGAD00001001473, EGAD00001002460 and EGAD00001002508. Source data are provided with this paper.

Research involving human participants, their data, or biological material

Policy information about studies with human participants or human data. See also policy information about sex, gender (identity/presentation), and sexual orientation and race, ethnicity and racism.

Reporting on sex and gender	Sex and gender were not considered in this study design.
Reporting on race, ethnicity, or other socially relevant groupings	Race, ethnicity or other socially relevant groupings were not considered in the study design.
Population characteristics	Age was not considered during data selection (extremes were avoided). Only healthy samples were included.
Recruitment	Datasets were included based on the availability of isolated cell types, fraction estimation (for in vitro datasets) and healthy conditions.
Ethics oversight	No ethics approval was necessary for this study.

Note that full information on the approval of the study protocol must also be provided in the manuscript.

Field-specific reporting

Please select the one below that is the best fit for your research. If you are not sure, read the appropriate sections before making your selection.

☒ Life sciences ☐ Behavioural & social sciences ☐ Ecological, evolutionary & environmental sciences

For a reference copy of the document with all sections, see nature.com/documents/nr-reporting-summary-flat.pdf

Life sciences study design

All studies must disclose on these points even when the disclosure is negative.

Sample size	As many publicly available samples as possible were included, based on the inclusion criteria. The resulting number of samples was sufficient for reference and in silico mixture building as well as subsequent deconvolution performance assessment.
Data exclusions	Two samples that were expected to be of low quality were excluded (outliers based on methylation profiles).
Replication	Benchmarking of deconvolution was performed for several data formats (array-based and sequencing-based) and on in silico, in vitro and in vivo data.
Randomization	This was not relevant for the study, as all samples were healthy.
Blinding	This was not relevant for the study, as all samples were healthy.

Reporting for specific materials, systems and methods

We require information from authors about some types of materials, experimental systems and methods used in many studies. Here, indicate whether each material, system or method listed is relevant to your study. If you are not sure if a list item applies to your research, read the appropriate section before selecting a response.

Materials & experimental systems

n/a	Involved in the study
☒	☐ Antibodies
☒	☐ Eukaryotic cell lines
☒	☐ Palaeontology and archaeology
☒	☐ Animals and other organisms
☒	☐ Clinical data
☒	☐ Dual use research of concern
☒	☐ Plants

Methods

n/a	Involved in the study
☒	☐ ChIP-seq
☒	☐ Flow cytometry
☒	☐ MRI-based neuroimaging

Plants

Seed stocks	*Report on the source of all seed stocks or other plant material used. If applicable, state the seed stock centre and catalogue number. If plant specimens were collected from the field, describe the collection location, date and sampling procedures.*
Novel plant genotypes	*Describe the methods by which all novel plant genotypes were produced. This includes those generated by transgenic approaches, gene editing, chemical/radiation-based mutagenesis and hybridization. For transgenic lines, describe the transformation method, the number of independent lines analyzed and the generation upon which experiments were performed. For gene-edited lines, describe the editor used, the endogenous sequence targeted for editing, the targeting guide RNA sequence (if applicable) and how the editor was applied.*
Authentication	*Describe any authentication procedures for each seed stock used or novel genotype generated. Describe any experiments used to assess the effect of a mutation and, where applicable, how potential secondary effects (e.g. second site T-DNA insertions, mosiacism, off-target gene editing) were examined.*
